# Mobilising Affect for Public Art: Affective practices in voluntary organising

**DOI:** 10.1177/01708406241273828

**Published:** 2024-09-16

**Authors:** Christina Lüthy

**Affiliations:** Lund University, Sweden

**Keywords:** affect theory, attunement, coalitional moments, contemporary art, ethnography, materiality, practice theory, voluntary organising

## Abstract

Voluntary organising frequently relies on affective intensities to direct organisational efforts. However, it is not well understood how these intensities are cultivated across time and different contexts to engage and coordinate heterogeneous actors. By applying a practice approach to affect, this paper proposes the concept of affective practices to theorise how affect is mobilised in materially driven (inter)actions to shape actions and relationalities around organisational goals. The analysis of ethnographic data from a long-term public art project reveals that four affective practices – enticing, envisioning, attending and asserting – are pivotal to sustaining the distributed process of voluntary organising. The sense of fascination, enthusiasm, care and discomfort that these affective practices mobilise instigates participation, support, acceptance and compliance from diverse partners, volunteers and the local public. Contributing to the affective turn in practice theory, the paper theorises how affective processes are cultivated as situative accomplishments in an ongoing and translocal organisational process, highlighting the important role played by the vibrant presence of matter in affective practices. Additionally, the study expands our understanding of how an interplay of affective intensities engages and aligns diverse individuals and groups in voluntary organising by fostering coalitional moments in the organisational process.

## Introduction

The recent turn towards affect in organisation studies has called attention to the fact that organisational life is suffused with a flurry of affective intensities (Fotaki, Kenny, & Vachhani, 2017). Affects are understood as bodily sensations ‘that connect and repulse subjects’ and impart movement, priming capacities for action and relationality (Bruce & Homan, 2018, p. 277; Clough, 2007). Particularly in voluntary organising, where collaboration and coordination are shaped by informal and relational dynamics rather than contractual agreements, affect is a powerful force that orchestrates organisational processes, directing commitment and co-orientation in fluid collectives (Endrissat & Islam, 2022) and fostering purpose and allegiance around shared goals (Reinecke, 2018; Vachhani & Pullen, 2019).

Studies tracing how affective processes support voluntary organising have highlighted that affects are cultivated and circulate in a particular ‘time–space’ (Marsh & Śliwa, 2022, p. 480), for example, in a street protest (Marsh & Śliwa, 2022; Reinecke, 2018) or at a community-oriented event (Endrissat & Islam, 2022; Ruebottom & Auster, 2018). These studies also point to the fragility of sustaining voluntary organising through affective processes (Vidolov, Geiger, & Stendahl, 2023) because affective intensities tend to ‘evaporate’ (Resch & Rozas, 2024; Waters-Lynch & Duff, 2019, p. 12). However, little is known about how voluntary organising can cultivate affective intensity across different organisational contexts and over time. Moreover, most studies focus on how voluntary organising assembles around one key affective dynamic to engage core contributors (Vachhani & Pullen, 2019; Vidolov et al., 2023), for example, by moving them from fear to laughter (Marsh & Śliwa, 2022). Conversely, scant attention has been given to how voluntary organising might rely on inspiring multiple forms of affective relationality to engage and tie heterogeneous publics and interests to organisational goals. Considering that eliciting affective intensities across time and space is critical to holding together precarious processes of voluntary organising, and acknowledging that these organisational processes usually depend on garnering support from diverse individuals and groups, it is critical to develop an understanding of the mobilisation of affect that can account for the heterogeneity, translocation and ‘ongoingness’ of affective processes in voluntary organising.

To that end, I suggest in this paper to move away from studying affects primarily as sited constellations that are cultivated in a specific time–space of voluntary organising. Instead, I propose tracing affects as patterned actions that are enacted across spatial and temporal dimensions of organisational processes through embodied ways of ‘doing’ and ‘saying’ and their entanglement with different materialities. Building on a practice approach towards affect (Gherardi, 2017a; Kuhn, Ashcraft, & Cooren, 2017; Reckwitz, 2017; Wetherell, 2013), I argue that, although all practices have an affective dimension (Reckwitz, 2017), we can identify a class of practices that have a particular focus and intent on evoking affect to shape action and relationality around organisational objectives.

To explore the mobilisation of affect in voluntary organising, I turn to contemporary forms of artistic organising that produce public works of art as ongoing participative accomplishments (Bishop, 2012; Bourriaud, 2010; Jackson, 2011). Artworks such as *City of Change* by the International Institute for Political Murder combine temporary poststudio visual art and performance to create and facilitate open-ended processes of collective engagement in public spaces (Beyes, 2015). Because the artists provide a framework for creative interaction rather than a finished aesthetic experience, they must motivate various publics to contribute to and engage with the artistic idea through participation. Moreover, the organisation of these artworks depends on the willingness of diverse stakeholders to accommodate the artistic process and give it a habitat. Different scholars have observed that the organisational accomplishment of this genre of public art is predicated on artists’ ability to catalyse, (re)configure and intensify affective intensities (Bruce & Homan, 2018; Holm & Beyes, 2022; Svejenova, Strandgaard Pedersen, & Vives, 2011). Therefore, they are a particularly interesting empirical context for studying how affect as a ‘technology of organising’ (Endrissat & Islam, 2022, p. 1042) is elicited in situated activities as a way to foster and shape the voluntary engagement of a meshwork of different contributors across various temporal and spatial points in the organisational process.

The empirical data in the current study were gathered by following the work of an artistic enterprise and one of its long-term participative public art projects over a period of three years. The artists highlight that organising is all about working with intensities because they continuously need to engage diverse individuals, groups and institutions in supporting Bignik, a growing modular picnic blanket that covers large areas of public space and aims at fashioning a new form of conviviality and co-creation in public life. Conducting an ethnography *of* and *with* affect (Gherardi, 2019; Kuhn et al., 2017), I attuned to how such intensities were cultivated in the relational space of recurrent patterns of (inter)actions and how they moved volunteers, partners, residents and local institutions towards relating and attaching to the artistic project in ways that served the organisational process. The analysis carves out four affective practices – enticing, envisioning, attending and asserting – that mobilise affect to activate and sustain the process of voluntary organising and emphasises the important role that materialities play in their enactment. The findings show how these affective practices allure people into situated and incidental acts of engagement and support (enticing) and infect participants with a purposeful enthusiasm to pursue the artistic project (envisioning). Moreover, the findings also shed light on how affective practices address friction in the process of voluntary organising by fostering a sense of mutual care and acceptance (attending) or, on the contrary, by frustrating partners into abandoning interests and ideas that are not aligned with the core artistic idea (asserting).

With the present paper, I make two contributions. First, I contribute to the ‘affective turn’ (Clough, 2007) in practice theory by introducing the concept of affective practices, thus expanding a practice theoretical understanding of affect in organisation studies (Gherardi, 2017a; Kuhn et al., 2017; Reckwitz, 2017; Wetherell, 2013). Affective practices are patterns of (inter)action that have the explicit aim of evoking affective intensities for organisational purposes. The concept of affective practices allows us to theorise how affect is mobilised in voluntary organising as an ongoing and translocal accomplishment, emphasising that sustaining voluntary organising is critically dependent on the ongoing (collective) adoption, re-enactment and circulation of these affective practices. Moreover, the current study also extends organisational research on ‘vital materiality’ (Bell & Vachhani, 2020) and ‘affective objects’ (Vidolov et al., 2023, p. 5) by showing how the vibrant presence of diverse everyday matters operates as an ‘affect generator’ (Reckwitz, 2017, p. 123) enabling affective practices to project new organisational interests, promises and threats under different circumstances and towards different publics. As a second contribution, the paper contributes to the literature discussing how affective intensities coordinate voluntary organising. The study shows how four distinct positive and negative affective intensities elicit ‘coalitional moments’ (Bruce & Homan, 2018, p. 226) that engage and align the commitment, support and compliance of diverse participants, partners and stakeholders by priming ongoing relational processes and coaxing them towards organisational goals. Furthermore, I reflect on how the situative evocation, compiling and balancing of intensities, and the resulting coalitional energy, also produce subtle forms of domination and infraction that can leave intrusive affective traces in the organisational process.

## Theoretical Background

### Affective relationality and voluntary organising

Over the past decade, affect theory has garnered increasing attention in organisation studies, here following a broader turn to affect in the humanities and social sciences (Clough, 2007; Gregg & Seigworth, 2010). Although affect has often been depicted as an autonomous and unpredictable force that escapes and destabilises the normativity of organising (Beyes & De Cock, 2017; Massumi, 2002), recently, there has been a wave of studies showing how affect circulates and suffuses fluid collectives with relational and organisational capacities (Endrissat & Islam, 2022; Keevers & Sykes, 2016; Marsh & Śliwa, 2022; Pouthier & Sondak, 2021; Resch & Rozas, 2024). Critical organisation studies scholars have emphasised the ‘relational organising power’ (Angerer, 2015, p. 115) of affect, pointing towards the important role that affect as relational intensity plays in political, collaborative and artistic forms of voluntary organising (Endrissat & Islam, 2022; Marsh & Śliwa, 2022; Vachhani & Pullen, 2019; Vidolov et al., 2023). Research on affective processes in voluntary organising is underpinned by resonating as well as contrasting conceptualisations of affect, mirroring the multidisciplinary history of affect theory and the debates and cross-fertilisation between different traditions that have shaped it. Although affect escapes any singular definition, different conceptualisations share the idea of affect as a bodily sensation, energy or intensity that affords action and relationality, prompting people to participate in collective efforts and providing a ‘glue’ that holds voluntary organising together in the absence of formal structures.

Deleuzian-inspired affect theorists have often underscored the difference between affect and emotion (Massumi, 2002; Thrift, 2008), conceiving the former as an untamed visceral force that escapes discursive representation while denoting the latter as a culturally named and domesticated feeling. However, feminist scholars (Harris & Ashcraft, 2023; Hemmings, 2005) have questioned this strong dichotomy, approaching affect and emotion as poles rather than planes of difference in phenomena. Their critique highlights that affect as a ‘materially felt social relation’ (Kuhn et al., 2017, p. 61) reverberates in the texture and tone of voluntary organising, for instance, as a sense of urgency, excitement or community that circulates and motivates people to contribute to and co-orient in hackathons as fluid collectives (Endrissat & Islam, 2022). At the same time, affect can also cascade, move and be traced in emotional representations of affective experiences, for example, when members of a Polish artistic collective recount how their fear vanished in a playful and absurd street protest and engaged the public in forms of nonconfrontational resistance (Marsh & Śliwa, 2022). Thus, affect theory draws our attention to how affective intensities reverberate in felt experiences and understands these intensities as social processes that prime and (un)do tendencies of relating and social ordering in voluntary organising. Moreover, by questioning the idea of a contained body and the individual who resides in it, affect theory emphasises how voluntary organising emerges and is sustained as porous selves are moved by the human and nonhuman world that surrounds their sensate bodies (Blackman & Venn, 2010).

Most studies focus on how the affective intensities that enable voluntary organising circulate in a particular time–space, moving participants, for example, through the vibrant atmosphere and feelings of connection and intimacy with others at public protests (Marsh & Śliwa, 2022; Reinecke, 2018), through carefully curated and temporally intensified ‘buzz’ (Resch & Rozas, 2024, p. 7) in community innovation spaces or through personal stories shared on online platforms (Endrissat & Islam, 2022, p. 1024; Vachhani & Pullen, 2019; Vidolov et al., 2023). These empirical accounts underline how voluntary organising engages people through affects that circulate and intensify at specific physical and virtual sites, but also easily collapses if affective processes are not sustained (Vidolov et al., 2023; Resch & Rozas, 2024). Arguably, stoking affect in a focal time–space is not sufficient to maintain organisational processes because voluntary organising typically spans multiple locations and occasions and needs to engage people beyond single sites and key events. However, few studies have examined how affective intensities are mobilised and shape dispersed and ongoing processes of voluntary organising.

At the same time, the literature has predominantly emphasised how affective intensities coordinate voluntary efforts and commitment to organisational causes and ideas by unsettling, connecting and orienting people around a key axis of affective movement and relationality. For example, Vachhani and Pullen (2019) reveal how transfiguring shame and outrage into affective solidarity fuels voluntary organising in feminist initiatives. Endrissat and Islam (2022) show that channelling a collective sense of achievement into urgency and excitement engages and orients participation in co-creation events, while Vidolov et al. (2023) analyse how a patient movement is driven by the affective dissonance emanating from the interconnected evocation of pain and hope. In all these examples we see how voluntary organising is driven through one core conversion of affect. However, they take little notice of how a multiplicity of distinctive affective relationalities might be cultivated and combined to engage and coordinate voluntary efforts. This is surprising given that many forms of voluntary organising rely on support, endorsement or, in some cases, the mere acceptance of heterogeneous publics with different and occasionally shifting affinities for being moved and engaged along a certain affective axis. The struggle to account for the heterogeneity, translocation and ongoingness of affective processes in voluntary organising highlights the need to go beyond studying affects as sited constellations and develop a more granular and dynamic understanding of the mobilisation of affect in voluntary organising.

### Practices and the mobilisation of affect

A practice lens to affect appears particularly useful for expanding our understanding of ‘how affect emerges’ (Fotaki et al., 2017, p. 8) and is sustained across complex organisational processes. Practice theorists emphasise that practices as embodied, material and directed patterns of doings and sayings are constitutive of organising (Schatzki, 2006), enacting both large cultural spheres (e.g. shopping; Gonzalez-Arcos, Joubert, Scaraboto, Guesalaga, & Sandberg, 2021) and structuring the minutiae of organisational processes and encounters, for example, through attention (Nicolini & Korica, 2021). Over the past decade, practice scholars have developed a growing interest in affect theory (Ashcraft, 2021; Gherardi, 2017a; Kuhn et al., 2017; Reckwitz, 2017), demonstrating that practice approaches are fruitful for studying how affect emerges and what it ‘does’ in and to the relational activities of organising.

Coming from the perspective of critical discursive psychology, Wetherell (2012, 2013) was one of the first to point out that affect should be traced in the flows and patterns of situated activity. Referring to Sedgwick (2003), she highlights that affect research needs to pay attention to the embodied ‘texture’ of assemblages and articulations of practices across multiple modalities and the ‘middle ranges’ of agency that they configure. Adopting a relational and material ontology (Kuhn et al., 2017), I draw on practice scholars who emphasise how affects are experienced and generated within the ‘assembling of bodies, activities and materialities’ in situated activity (Gherardi, 2017a, p. 348; Keevers & Sykes, 2016; Kuhn et al., 2017; Reckwitz, 2017). This work directs our attention to the complex gathering of artefacts, spaces, words, gestures, ideas, expectations and sensations that are entangled with one another in practice and how they forge a relational space in which affect emerges and moves. Although the practical accomplishment of such entanglements is fragile and dynamic, moving bodies in a series of (uncertain) affective becomings (Gherardi, 2017b), it is also (re)productive of social order, creating and sustaining ways of ‘doing things together’ (Katila, Kuismin, & Valtonen, 2020, p. 1311), for example, upbeat collaboration at entrepreneurial idea workshops.

Practice theorists have argued that all social practices have an affective dimension that is experienced and actualised through the enactment and responsiveness of the participating human and nonhuman bodies (Gherardi, 2017c; Reckwitz, 2017). At the same time, Reckwitz (2017) and others (Gherardi, 2017b; Wetherell, McConville, & McCreanor, 2020) have suggested that we can identify practices that have a particular ‘intent’ and ‘emphasis’ on affecting the bodies that participate in the relational space of a practice, for instance, social media ‘call-out’ practices and their attempt at shaming people and behaviours. Reckwitz (2017) highlights that practices with such an affective intent often draw on things as affect generators and distinguishes between material artefacts (e.g. objects and architecture) and imaginary-semiotic artefacts (e.g. texts and images), which transport signs and imaginations. His ideas resonate with research in organisation studies that emphasises how materialities, understood as things or entities that ‘matter’ (Ashcraft, 2021), can function as generators through the ‘vibrant’ (Bennett, 2010) presence they assume in our embodied and sensuous relationship with them, be it in our tactile encounter with objects (Vachhani, 2013), as colour that we ‘see-feel’ (Beyes & De Cock, 2017) or as speech that moves us through the physicality of sound as much as the visuality of all-caps text (Ashcraft, 2021). In practices, materialities help mobilise affects by stimulating bodily feelings of (dis)pleasure and (de)activation directed towards specific ideas, objects, actions and people (Reckwitz, 2017).

By locating affective processes in directed patterns of activities, a practice approach to affect allows us to explore how voluntary organising can cultivate and sustain affective intensities over time and across organisational contexts through the recurring enactment of certain sayings and doings and how they draw on the vibrant presence of materiality to move bodies. We can trace how practices mobilise affect in sensory cues, such as changes in facial expressions and gestures, tone of voice and posture, resonating atmospheres and feelings, or we sense them lingering in emotional expressions and actions stirred up by affective experiences. Moreover, a practice approach calls us to pay close attention to what affects ‘do’ to voluntary organising – what actions and forms of relationalities they prime – and allows us to study in a granular and dynamic way how different affective relationalities are enacted in voluntary organising to foster and coordinate support and engagement.

## Methods

### Following the practices of artistic organising

Scholars have frequently adopted ethnographic approaches to explore practices as skilful sociomaterial accomplishments (Gherardi, 2019; Nicolini & Monteiro, 2017). My investigation of the activities and organisational processes of the Swiss art enterprise Atelier für Sonderaufgaben [Studio for Special Tasks] (AfS) was driven by an interest in understanding how affect configures voluntary organising and how affects are mobilised in situated organisational activities. Founded and directed by brothers and concept artists Frank and Patrik Riklin, the AfS has become renowned for creating art ‘in the middle of society’ in collaboration with different institutions and organisations. Their well-documented and internationally discussed artworks, which interweave sited interventions with long-term participative processes, are organised around a multiplicity of different actors and publics that engage in the artistic creation of ‘new social realities’.

**Figure 1.**
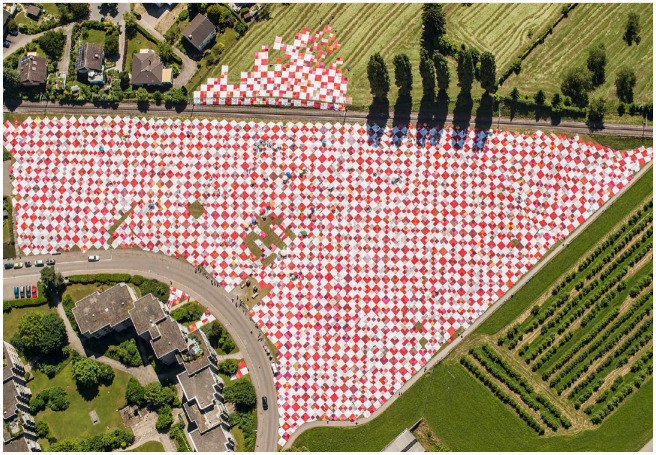
Bird’s eye view of the assembly of the blanket in 2017.

As a researcher, I followed the work of AfS and the evolution of several existing and emerging artistic projects over a three-year period. As is typical of fieldwork, I iterated between different roles and forms of participation that were shaped by the logics of the field (Bruni, 2006). Initially, I observed the meetings and day-to-day activities inside and outside the studio as a bystander. As the fieldwork and situations in the field progressed, I became organically more involved in organisational processes. I was asked to reflect on project processes as a collaborator, to provide feedback on concepts, plans and prototypes and to help organise and set up materials while experiencing and contributing to the artwork as a participant and spectator. Although the importance of practices for mobilising affect emerged from the entirety of the fieldwork, I chose to focus the analysis on Bignik as a particularly illustrative project (see Figure 1).

Bignik is a long-term participative art project that the AfS has been organising since 2011 in partnership with the communal development agency (CDA) of Eastern Switzerland. The utopian vision for the project is to create a modular and constantly growing picnic blanket for the entire region of Eastern Switzerland, eventually comprising one module per inhabitant. Throughout the year, donations of red and white fabric are collected in different municipalities. At home and during public sewing events, volunteers from the region sew cloth into joinable square modules. Once a year in summer, the ever-growing amorphous Bignik blanket is assembled in a different town in Eastern Switzerland. Taking over fields, streets and squares, the project invites people from all over the region and beyond to participate and spend time together on the blanket. Despite running for several years, the project’s organisational process has remained fragile, depending on the engagement, acceptance and ongoing support of a complex coalition of key partners, volunteers, residents, participants, local authorities and institutions.

### Embodying an ethnography of and with affect

#### Data collection

To study the affective dimension of organising, researchers have suggested methodological approaches that build on deep and embodied engagement with a research context through fieldwork (Keevers & Sykes, 2016), supported by video data (Katila et al., 2020), interviews (Gherardi, Murgia, Bellè, Miele, & Carreri, 2019) and archival data (Marsh & Śliwa, 2022). The researcher’s bodily capacity to affect and be affected and enter resonant relationships with the human–nonhuman world is understood as a central epistemic instrument in the research process (Gherardi, 2019). Engaging in a style of fieldwork that I qualify as an ethnography *of* and *with* affect, I drew on attunement as a central methodological tool to sensitise my body to ‘energetic reverberations of practice and ride along where they might be going’, sensing ‘the sociomaterial relations in bloom’ (Kuhn et al., 2017, pp. 62–63) and ambiguous promises and threats emanating from them. Attunement enabled me to trace affects as ‘flows of imitation’ (Thrift, 2008, p. 237) that invite us to align our feelings and bodies as practices unfold.

During my fieldwork, I observed and participated in everyday discussions, engaged in reflection and contributed to the project by attending planning meetings and information events, as well as joining public sewing sessions and blanket assembly. Overall, 33 days of fieldwork revolved to varying degrees around the organisation of the Bignik. In the field, I organised my attunement around a series of sensitising questions inspired by practice approaches and affect research (see Table 1) (Gherardi, 2019; Nicolini & Monteiro, 2017; Vannini, 2015). These questions informed my registering and recording of how affect as relational intensity worked in practice. For instance, I documented how I was gripped by a sense of allure when the artists showed images of the storehouse brimming with colourful fabrics and how this fascination captivated other collaborators in the meeting as they reacted to the images and touched the fabrics circulated by the artists. I used pictures and videos to capture the material and spatial settings in which these activities and intensities unfolded to complement my field notes.

**Table 1.** Sensitising questions guiding the attunement in the field.

Focus	Ethnography of and with affect: Sensitising questions
Doings	What is being done and through what kinds of movements, gestures and rhythms? Who is involved in and affected by these ‘doings’?
Sayings	What is being said and with what kind of intensity? What kinds of concerns, ideas or experiences are being expressed and invoked?
Materialities	What sensations (sounds, smells, textures, colours etc.), artefacts (images, objects) and spaces enfold the doings and sayings?
Affect	What intensities (moods, atmospheres, feelings, vibrations) are palpable in the situation? How do they relate to the doings, sayings and materialities present?
Relations	What kinds of (bodily) relations with humans and nonhumans are enacted? How are these relations moved and underpinned by affective intensities?

As a research approach, attunement has its own limitations that must be acknowledged. The researcher’s abilities to sense differences and movement and resonate with ‘vibrations’ (Ash & Gallacher, 2015) in the field are continuously refracted through the prism of her own bodily history and presence. Hence, I tried to be aware of my susceptibility to certain affective transitions and intentionally attuned myself to moments of indifference or dissonance that surrounded re-emerging affective patterns. Two group interviews with 13 participants and seven individual interviews lasting between 34 and 157 minutes as well as 19 short field interviews with individuals and groups lasting between 2 and 13 minutes helped decentre the fieldwork from my own embodied experience. The reflections shared by artists, collaborators, participants and the public during interviews and fieldwork provided a basis for detecting the affective reverberations of practices in the experiences of others. The use of documentary materials such as media reports, meeting minutes, concept papers and video documentation enabled me to track and contextualise the intensities I sensed in the broader history of the project. Triangulating my embodied experience with the materialisation of other voices and traces kept the ‘data on the move’, intensifying and complicating my own ‘sensing of’ and ‘becoming with’ practices and the intensities they stir in the field (Gherardi, 2019, p. 753) while increasing the robustness of the data and analysis.

#### Data analysis

I adopted an iterative interpretive and affective analytical approach that sensitised me to sticky affective patterns and enabled me to gradually interpret the embodied processes reflected in the empirical material (Gherardi, 2019; Kuhn et al., 2017; Van Maanen, Sørensen, & Mitchell, 2007). By reading and annotating the field notes and interviews, I recognised that a series of cognate affective intensities related to fascination, enthusiasm, care and discomfort were critical in moving diverse individuals, groups and institutions towards engaging with and supporting the artistic project and its goals (Gherardi, 2019; Van Maanen et al., 2007). To analyse how these affects were mobilised across time and space and shaped the process of voluntary organising, I started crafting a series of 12 vignettes around episodes where the emergence of these intensities was particularly palpable by revisiting not only my field notes but also the pictures and videos I took during the fieldwork. In these vignettes, I focused on my body as a ‘point of contact’ (Bell & Vachhani, 2020, p. 685; Gherardi, 2019; see also Stewart, 2007) to interweave snippets of dialogue, temporal flows of actions and relations, shifting bodily expressions, prominent objects and settings, spatial atmospheres and kinaesthetic experiences into rhythmic and textured accounts.

The (re)writing and interpreting of these vignettes were central to the analytic process. Drawing on a multidisciplinary literature (e.g. Bennett, 2001; Komporozos-Athanasiou & Fotaki, 2015; Meissner, 2014; Sage, Vitry, & Dainty, 2020) that explores how affective relationality emerges between different bodies, I began to identify how affects were mobilised in patterns of sayings and doings that drew on specific materialities (e.g. things, words, shared rhythms and practices or ideas). I noticed, for instance, how the staging of wondrous or intriguing encounters with the fabrics kept enticing people into temporarily embracing and engaging in the project. I then coded the corpus of field data to trace the recurrent configuration and effects of these practices in other incidences, in the experiences of collaborators and participants and in the broader context of the project, such as in the accounts of participants who recalled themselves and others being drawn out of their everyday way of acting as they were struck by the colour and texture of the artistic process. Furthermore, I observed how these practices were enacted not only by the artists, but also by other collaborators, for example, when partners started using imaginative language when discussing the project with others. Eventually, through an iterative process of clustering, triangulating and interpreting my data sources, I discerned enticing, envisioning, attending and asserting as four practices enacted in the organisational process and analysed how they mobilised affective intensities through materially driven (inter)actions to move people towards relating to the artistic project in beneficial ways (see Table 2 in the Appendix). In the findings, I assemble snippets of vignettes, observations and voices from the field into an analytic text that evokes, tracks and interprets what I call *affective practices* and the way they shape and sustain the ongoing process of voluntary organising.

## Findings

The key findings revolve around four *affective practices* and how they are enacted across the organisational process to elicit distinct supportive relationships with Bignik. Although the analysis traces these affective practices mostly through separate incidences, they are frequently combined in the organisational process to strengthen or consolidate their effects.

### Enticing

The affective practice of enticing is enacted to draw participants, bystanders and collaborators out of their everyday inclinations. By instigating a sense of fascination and wonder, enticing incites them to embrace the artistic project and support it through temporary and situated acts of engagement.

Transporting the sea of fabric from the car to the sewing station next to the town hall, a sense of awe overcomes me. A first-time collaborator sighs briefly. A hint of amazement resonates in her voice as she stares at a large ball of cloth: ‘I have never seen so much fabric!’ Around us, people are visibly intrigued by the vibrant materiality sprawling in the public space. They gaze puzzled at the colourful mess that stands out against the grey town square, stop to take a picture or run their fingers over the colourful material. A passer-by shakes his head with a big smile: ‘You are still here!’ Inquisitively, people study the flyers and giant prints of the blanket stuck to the walls, look transfixed at the video projection of the fabric collection or ask me questions about the project. Some sit down to sew a module. (vignette 9)

In the vignette, we see how the artistic project and the way it is staged in public space captivates passers-by, volunteers, partners and participants, luring them to step out of their routines for a moment to explore and interact with the project. When setting up the various elements of the sewing station, the artists carefully consider how their arrangement might foster surprising encounters and invoke a sense of wonder and intrigue that is inviting. They discuss, for instance, how to position the sewing machines and large bolts of cloth against the glass entrance of the town hall to create an interesting contrast or how to assemble the enormous pile of fabrics on a cart so that its exuberance looks uncanny and unruly yet aesthetic and welcoming. The different colours, textures and rhythms that the sewing process brings to the public space and the way it materialises the gargantuan scale and longevity of the project evoke a sense of wonder that incites people to embrace the artwork and its presence in everyday life, even if they do not fully understand it. The fascination manages to override feelings of annoyance and prompts small acts of engagement and contribution. The AfS continuously tries to harness the power of such ‘charming’ disturbances, often claiming that ‘we break in so others can break out’, that is, forging unexpected encounters and interactions so people can let go of their everyday inclinations momentarily.

Enticing works by engendering both a pleasurable feeling of being swept along by surprise and a slightly uncanny feeling of being ‘torn out of one’s default sensory-psychic-intellectual disposition’ (Bennett, 2001, p. 5). It circulates between participants and residents who find themselves lingering at the sewing station, even though they should go back to work or catch a train, and are astonished by their own behaviour and that of others. A volunteer who has participated before and spontaneously decided to join today pauses with a quizzical expression on her face as she surveys the masses of fabrics, noting that she still finds it strange to create such an enormous blanket: ‘However, I guess it is art. Art doesn’t have to be sensible.’ She laughs in surprise and points towards the artists: ‘They do this with such seriousness that automatically you do it seriously, too.’ By enveloping people in a combination of delight and disturbance, enticing enables them to welcome and become charmed by the sewing process while temporarily dislocating them from their habitual attachments.

The vibrant presence of red and white fabrics plays a central role in the affective practice of enticing. By structuring the flows and rhythms of sensuous experience (e.g. disrupting everyday spaces with an element of unexpected colour and luminosity, creating soft and textured surfaces that are surrounded by an earthy smell of cupboards and attics) and the affective relations they invoke (e.g. the way they trigger a marvelling gaze or invite a curious touch, awakening a longing for playful creation), the fabrics produce a state of ‘interactive fascination’ (Bennett, 2010, p. 5) that attracts collaborators to embrace and engage with Bignik. A member of the steering committee remembers how chairpersons who struggled to relate to the project became drawn into the organisational process temporarily when the artists staged the project around haptic encounters with the fabrics: ‘On the one hand they conclude, “Those artists, they have a screw loose. That cannot work. They are odd.” On the other hand, they say, “Well, the overall idea isn’t that bad” and become enthralled with the sewing.’ The sensuous and relational capacities of the fabrics and the intensities that emanate from their ‘material vibrancy’ (Bennett, 2010, p. xiii) grip collaborators and publics, drawing them out of their usual thoughts and actions into pleasurable encounters and interactions with the artistic project.

### Envisioning

Although enticing is often successful in captivating people into fleeting engagements and a temporary embrace of the artistic project, the organisational process also needs to infect people with a more purposeful desire to realise and participate. Envisioning is enacted to elicit a sense of excitement and enthusiasm around the artistic vision that forges an affective attachment of collaborators, participants and partners to materialise the artistic project.

People sit on improvised rows of chairs in the centre of the municipality’s foyer, listening intently, as Frank presents the plan for assembling the Bignik. He introduces the project as a ‘growing artwork’ that is about building ‘unusual communities’ while projecting film fragments and stunning photos of previous assemblies. The man next to me smiles enthusiastically. As he shows an aerial visualisation of the assembled blanket in the town, Patrik jumps in: ‘Bignik is like a fluid that is emptied in the streets around here.’ A sense of excitement grips me as I imagine covering alleys and squares with fabrics. Frank emphasises that everyone living here becomes an ‘accomplice’ in the project and that, together, they need to find ‘unorthodox solutions’. After the event, people’s voices are eager while they discuss and anticipate the project and how they could contribute. Reviewing the next steps, the mayor beams and jokes: ‘The Bignik virus has caught me!’ (vignette 7)

The vignette elicits how the project presentation creates a sense of excitement and zest to contribute to and materialise the artistic project among the mayor and other collaborators and residents at the information event. Talking about the project, AfS invokes the blanket as a ‘fluid made from fabric’ or ‘flexible paint’ while showing images and visualisations of coloured streets, inviting the audience to imagine becoming part of the assembly as a sensual experience of appropriating and transforming everyday spaces. When AfS holds the improvised town hall meeting in the middle of the foyer and frames the organisation of the Bignik around ‘becoming accomplices’, finding ‘unorthodox solutions’ and creating ‘unusual communities’, it conjures an imaginary of art as a collective endeavour that creates an experimental and communal site for all involved. Enthusiasm is palpable in the room after the event, the evocative presentation fostering a desire in the audience to make the project happen. A local artist points out that she usually does not like such large projects but that the talk made her think about how she could become involved because ‘it is important to create things together’. The mayor observes that AfS is skilled at turning people into devotees and provoking enthusiasm and loyalty around the project. Discussing her own bodily experience at the information event, she notes, ‘When they [the artists] tell it like this, you are just hooked.’

Envisioning operates by crafting imaginaries, that is, new and creative ‘word images’ (Komporozos-Athanasiou & Fotaki, 2015, p. 327), that foster excitement and anticipation by shaping both what the artistic project represents and the affective meanings it can offer to different collaborators and partners. Before important meetings, the artists often spend hours honing the exact wording and discussing how images, objects and the spatial setting could support this ‘mental cinema’, as they sometimes call it. The sense of excitement that these imaginaries evoke is contagious. It ‘infects’ people who hear about the project from the artists and are grasped by the enthused voices and glowing faces of other residents, participants and collaborators who eagerly anticipate the project and want to contribute to it. A collaborator notes, ‘[The two brothers] have this visionary imagination that can initiate social dynamics.’ The imaginaries help create new connections between the psychical and the social, giving polyvocal meanings to the artistic project that activate intersubjective desires and affective attachments oriented towards its organisational process.

The evocative presence of language plays a key role in the affective practice of envisioning. It builds on what Rancière (2007) calls ‘literarity’, which is the capacity of language to instigate new desires, mobilise other interests and emphasise different problematisations. By forging connections between people, materialities and systems of signification, language can articulate new scopes of relationships. For example, the term ‘accomplices’, which AfS frequently uses to address audiences, prompts people to become co-creators and allies of this artwork. At the same time, the term articulates a new problem: the necessity and importance of their complicity in the project. The imaginative quality of the language and terminology used by AfS also works when presented in written form on social media or in the press. However, it seems to be particularly effective when experienced in an embodied way in resonance with other words, spaces, objects and actions surrounding it. For example, the words ‘unorthodox solutions’ are transported by the conspiratorial voice of the artists, accompanied by playful questions and suggestions on how to deal creatively with the closed streets and embedded in the ad hoc use of space. An audience member at the information event shares: ‘I have read about it and seen images before, but now there is more excitement behind it, after hearing it live.’

### Attending

Mobilising a sense of wonder or excitement cannot always override feelings of alienation or tension among people who are involved in or impacted by the project. Members of the CDA and the steering committee mention that at times they felt overwhelmed or even threatened by the scope, disruptiveness and complexity of the project and wanted to stop it. As an affective practice, attending is enacted to move partners, participants and audiences towards accepting difficult or inconvenient aspects of the project by instigating a sense of mutual care and respect.

I sense a tenseness in the sombre dining room as the restaurant owner greets us stiffly and joins the table. The artists want to know whether the seating arrangement works and compliment his food. A flicker of approval crosses the owner’s face as the artists start asking about the restaurant’s history and business situation. Gazing sternly at the large map showing the potential assembly of the blanket around his establishment, the owner fends off the project: ‘The road needs to stay open. I will lose many clients if they can’t drive here.’ Listening carefully to his objections, the artists suggest that they could create ‘slippers’ for car tyres so that visitors can drive to the restaurant without harming the blanket. The restaurant owner looks sceptical but acquiesces. As he slowly starts engaging with the artists’ ideas, I can feel my body relaxing. (vignette 8)

The vignette shows how the AfS engages with an agitated restaurant owner who told them with barely concealed exasperation that they are ‘out of their mind’ after hearing that the roads to his establishment might be blocked by the blanket. By taking the time to explore the owner’s relationship with the restaurant, the history that shaped it, and his broader business approach, while also trying to find ways to accommodate his concerns, the artists instigate a sense of mutual care and respect. Although he is not entirely convinced by the suggested solution, he eventually agrees to allow Bignik to cover the streets leading to his property. The visit and attentive presence of the artists make the restaurant owner feel valued and seen. An earnest appreciation is palpable when the restaurant owner tells me, ‘It was important to discuss the matter: What do [the artists] imagine? Where are my needs? And it is always a communication thing: How does it take place? And it took place in the right way.’ The artists are aware that shared respect and caring relationships are necessary to ensure the collaborative realisation of the artistic project. Patrik emphasises, ‘I think you can convince many people only if you like them. When you are in constant conflict with them, then you cannot convince them . . . It takes a certain empathy. It takes love; then, it can work.’

Attending creates this sense of respect and care because it enacts what Haraway (2012) refers to as ‘response-ability’ – a care for and responsiveness towards shared (and, at times, involuntary) entanglements created in this case by the artistic project. It implies ‘a solidarity that is not based on proximity and similarity but on being-in-this-together’ (Meissner, 2014, p. 40) and affirms collaborators and publics as legitimate and important ‘others’ while asking them to tolerate aspects of the project that feel inconvenient or unsettling to them. When successful, it evokes a double movement in the organisational process. The artistic project materialises by tending to the concerns of important collaborators, partners, participants and publics while they also attune themselves to the artistic process. Alluding to this movement in her relationship with the Bignik project, the mayor says, ‘Sometimes, you have to put yourself into that world as well because there are those moments when you could say, “They [AfS] are completely crazy, right?”’

In the affective practice of attending, the material presence of shared ‘practice worlds’ (Sandberg & Dall’Alba, 2009, p. 1355) helps elicit this sense of respect and care among reluctant partners and collaborators. It creates a space for the pace, rhythm and expectations of different collaborators to converge, creating a platform for joint attunement to occur. At the same time, inhabiting others’ practice worlds communicates appreciation and respect for their concerns and lifeworlds. During lunch at the restaurant, the practice of dining out and the embodied relations created between the AfS and the restaurant owner enable the AfS to mirror the restaurant owner’s concern about being a good and accessible host. The owner appraises this: ‘It is easier to communicate with people and find solutions when you sit together here at the table, especially since it is happening in front of my door.’ After visiting a farmer inconvenienced by the project, the artists explain that they have offered to make hay for a day and brought a bottle of wine and pasta along with a Bignik module. By mirroring what is relevant to the farmer (making hay) and validating his efforts, AfS establishes the farmer’s lifeworld and concerns as relevant to the project: ‘This has smoothed over our differences. Now, he is open again, but we have to anticipate that it might be too much for him.’

### Asserting

To ensure that the core idea of the artistic project is not undermined in the process of exploring and accommodating the concerns of collaborators, partners and the public, attending is often enacted and paired with asserting as an affective practice. Asserting creates a sense of unease and frustration among collaborators for having ideas or interests that are not aligned with the artistic problematisation underpinning the project.

An apprehensive energy surrounds members of CDA as we travel to meet the artists for a strategy meeting. We gather in an old industrial complex where the fabrics are stored. Stuck to the lofty white walls are pictures from the project over the years as well as a printout of the very first project proposal. In the discussion, CDA introduces the concept of a ‘Smallnik’, where people can rent a module to have their own mini BIGNIK. The artists acknowledge that this is a side project that CDA cares about but quickly set boundaries. Patrik admonishes, ‘The tourism office can only rent out modules that they sewed themselves and each only one time. You cannot just use the blanket; you need to contribute to its growth.’ The CDA members squirm, looking abashed and frustrated. I feel the presence of the fabrics peeking out from the boxes around me, and a pang of guilt overcomes me for liking the idea. (vignette 4)

In the vignette, we see how a sense of discomfort and frustration emanates as CDA brings in ideas about how the project could be developed around consumer offerings. In the meeting, the artists very quickly limit or reject their suggestions by invoking the importance of the shared process of co-creation, which is materially present in the room in the form of pictures of the blanket’s ongoing growth, boxes of donated fabrics and the original project proposal. A sense of unease or even guilt envelopes collaborators for trying to move the project away from the core artistic idea. A member of the CDA justifies these tensions in the process: ‘We had to learn that certain things just aren’t up for discussion, because often it is those core aspects or like the basic idea – this simplicity of the Bignik. You almost destroy it when you start fiddling with that.’ By being responsive to the interest of CDA in Smallnik while also setting limits to its implementation, AfS safeguards the creative idea of Bignik as a participative artwork. A long-term partner points to this oscillation by referring to his own experience of collaborating with the AfS: ‘They are always searching and moving, and in certain aspects, you can truly think along and influence them, but in others, they are incredibly consistent. You could also call it stubborn but just very consistent.’

Asserting prevents the organisational process from watering down the artistic idea in the face of conflicting interests and agendas by instigating frustration and unease as ‘sad passions’ (Munro & Thanem, 2018, p. 63; see also Sage et al., 2020). These sad passions weave through the organisational history of the project and are tangible in the way that collaborators are tensing up as they anticipate difficult conversations around their ideas or when they admit almost apologetically that it can be physically and energetically draining to work with the artists. In response, some chairpersons distanced themselves from the project, and a CDA member revealed that, at some point, she had to take a break from the project. Sad passions help keep collaborators compliant with core ideas of the artistic project. Whereas frustration and disappointment stifle collaborators, discouraging them from pushing unsuitable agendas, unease or guilt prompt them to align their interests and efforts with the artistic problematisation. The mayor notes, ‘Sometimes, I get this tight feeling when I notice, “Oh, [the artists] want this,” and I see that there could be problems, and I don’t know how to make it work.’

The striking presence of the artistic idea as a ‘semiotic-imaginary artefact’ (Reckwitz, 2017, p. 124) plays an important role in the affective practice of asserting. Whereas envisioning evokes affective meanings of why people should engage in the project, asserting affirms the materiality of how the project works. Asserting anchors and ties the project to the affective force of core features of the artistic concept, such as the notion that Bignik thrives on contribution and co-creation rather than consumption, or the aerial image of a blanket taking over towns and fields, thereby creating a different aesthetic experience and space for encounters. The affective power of integral notions, images and experiences undergirds asserting as an affective practice by providing resistance, making collaborators ‘see-feel’ (Manning, 2008, p. 328) and assume responsibility for what could be lost or destroyed in the process of accommodating their ideas and interests. Invoking the notion of the artwork as a participative process makes the CDA receptive to the feeling that simply ‘using’ the blanket as a commercial offering would destroy the core idea that renders the project special. When the artists reject the restaurant owner’s request to keep his street open, aerial images and visualisations of the blanket support the affective practice of asserting by enabling the owner to sense how his claim might tamper with the project’s aesthetics and the social space it creates.

## Discussion

The main findings show how four affective practices are enacted to sustain voluntary organising in a long-term, multisited artistic project. Whereas enticing and envisioning intrigue and enthuse potential collaborators, thereby garnering their participation and support, attending and asserting produce feelings of respect and discomfort that make it possible to negotiate and transcend moments of tension that inevitably arise when engaging diverse partners, collaborators and the public. I first highlight how the concept of affective practices contributes to a practice theoretical understanding of affect and discuss its potential for theorising about how affective processes are sustained in voluntary organising across time and space. Moreover, I emphasise the important role that the vibrant presence of materiality plays in enacting affective practices. Second, I discuss how the study sheds new light on how affective intensities coordinate voluntary organising by showing how the situative mobilisation and compiling of different intensities fosters coalitional moments that engage and coordinate heterogeneous publics and partners.

### Sustaining voluntary organising through affective practices

First, the present study shows how affective practices as sites of encounter and becoming purposefully interpellate people into affective experiences, constructing significant and organised feelings of (dis)pleasure that direct attention, action and meaning to organisational ideas and goals in voluntary organising. Practice scholars have recognised that affective dynamics form the ever-present but rarely acknowledged backdrop of social practices (Gherardi, 2017a; Kuhn et al., 2017; Reckwitz, 2017). In the paper, I contribute to this affective turn in practice theory by showing that affect is not only an effervescent backdrop of practices, but how it can also be a crucial effect and intent of their organisational accomplishment. To highlight this, I suggest calling practices that aim to mobilise affective intensities for organisational purposes ‘affective practices’.

Affective practices allow us to theorise how affective intensities are cultivated, circulate and support voluntary organising not only in core time–spaces of the public art project, as Marsh and Śliwa (2022) call them, such as the assembly of the blanket, but also how this mobilisation of affect is taking place beyond such sited constellations and travels into the periphery of the organisational process. In the case of the Bignik, the ability to mobilise affect in everyday organisational interactions is critical to elicit and sustain the support and engagement of key collaborators, potential participants and partners. Arguably, this is also the case in many other forms of voluntary organising. For example, while hackathons thrive on the circulation, intensification and capture of affect during community innovation events (Endrissat & Islam, 2022), they likely also rely on the mobilisation of affect before and after the event to engage the support and resources of various stakeholders and community members that make hackathons possible.

The findings highlight that mundane materiality as an ‘affect generator’ (Reckwitz, 2017, p. 123) plays a critical role in enabling affective practices to mobilise affect under different circumstances and in different contexts by structuring resonant and sensuous – or in Clough’s (2010, p. 224) terminology, ‘infra-empirical’ – relationships in everyday interactions. Building on recent work emphasising the role of everyday materiality in shaping the affectivity of organisational practices (Katila, Laine, & Parkkari, 2019), encounters (Bell & Vachhani, 2020) and interactions (Ashcraft, 2021), the findings show how the four affective practices can move people by assembling around different material layers of the artistic project and by using their affective and relational capacities as vessels for projecting new interests, promises and threats (Kuhn et al., 2017). Enticing is fuelled by the sensuous lure of the blanket and the haptic encounters and aesthetic visuality it affords. Corroborating Bell and Vachhani’s (2020) new materialist conception of the affective pull of objects, enticing shows how materiality animates voluntary organising by attracting collaborators into new relationalities. Envisioning thrives on ‘symbolic activity as a kind of mattering’ (Ashcraft, 2021, p. 586) that elicits a desire through the aspirational force of imaginative language and the affective meaning it lends to the project, highlighting how such forms of mattering can affectively attach people to new problems and ideas (Rancière, 2007). Attending uses the presence of shared practice worlds to forge a common movement and affective space between different people (Katila et al., 2020; Keevers & Sykes, 2016) and thrives on the capacity of materiality to mirror interests and concerns. In contrast, asserting tunes into striking features of the artistic concept to establish it as a ‘semiotic-imaginary artefact’ (Reckwitz, 2017, p. 124) that elicits responsibility and subjection (Sage et al., 2020) and highlights that materiality not only mirrors but also affectively anchors collaborators in organisational interests and agendas through its vibrant presence.

Thus, the study also contributes to emerging research that explores the ‘vitality’ of matter in craft work, social work or communication (Ashcraft, 2021; Bell & Vachhani, 2020; Keevers & Sykes, 2016). The findings illustrate how diverse everyday matters beyond traditional objects can assume a vibrant presence in encounters with different collaborators and partners. Moreover, they emphasise that materiality animates affective practices and shapes relationality in multiple ways by affectively attracting, attaching, mirroring and anchoring heterogeneous organisational stakeholders. Public art projects are highly conducive to studying affective practices because contemporary artists are particularly reliant on and attuned to the affective capacities of matter and how it can be harnessed in practices to mobilise affect in organisational processes (Holm & Beyes, 2022). However, research also indicates that in other contexts voluntary organising thrives on organisers’ awareness of the affective potential of the ‘things’ that constitute their organisational sphere. Vidolov et al. (2023, p. 5), for example, show how patient movements are constituted through the circulation of ‘affective objects’ of pain and hope. Extending their insights, the present study indicates that the capacity of materiality to evoke pleasure and pain is not defined by objects per se, but rather by how material layers are invoked and become ‘vibrant’, hence affectively evocative in the assembling of an affective practice. Although it fell outside the scope of this analysis to systematically track how materialities exert their affective influence across multiple mediums of encounter, the analysis suggests that affective practices become more effective when they are experienced in a more embodied and sensorial way. This contrasts with Endrissat and Islam (2022), who argue that digital representations often generate more affect than actual physical encounters. Considering that voluntary organising increasingly moves and translates between online and offline spaces, additional research could trace how the transmediality and multimodality of affective practices shape affective flows and relations. Thus, the study calls for further explorations and integrative conceptualisations of how matters are enacted and circulated as ‘affective objects’ in organisational processes (Ahmed, 2010; Vidolov et al., 2023, p. 5).

Studies exploring the role of affective processes in voluntary organising have noted that affect, although perhaps quickly ignited, is also prone to dissipate, causing the organisational process to fade (Resch & Rozas, 2024; Vidolov et al., 2023). This is particularly obvious when studying how affect builds and evaporates at core sites and events of voluntary organising. However, the same can also be said for the mobilisation of affect through affective practices. In the artistic project, the affective pull elicited by enticing often does not extend far beyond the intriguing encounter, and even the energising grip conjured by envisioning can wane if it is not regularly rekindled and re-experienced. Supporting the idea that ‘affect produces stickiness through persistent contact’ (Kuhn et al., 2017, p. 176), a practice approach to affect theorises what other studies implicitly observe: affect as a relation of doing must be continuously re-enacted to sustain its organisational force. At the same time, affective practices draw our attention to the drivers and obstacles behind this re-enactment. In the empirical case of this study, the work of repetition is driven by the artists, who, as skilled practitioners, enact these affective practices routinely and reflexively. However, affective practices are also emulated by other collaborators and participants, albeit in a less conscious and intentional manner, indicating the collectivisation of affective practices as organisational technology. For example, residents reproach local authorities and insist on closing the main road, asserting and invoking the importance of the aesthetic coherence of the blanket. Affective practices provide us with a clearer conceptual understanding of both the potency and fragility of affectively driven modes of voluntary organising by showing how they thrive on the capacity of affective practices to circulate, catch and be adopted by a wide array of bodies, but also threaten to swiftly break down without people who are susceptible to their influence and capable of enacting them. Therefore, research should also focus on the conditions that are conducive to instituting the enactment of such affective practices (Holm & Beyes, 2022).

### Coordinating voluntary organising through fostering coalitional moments

Second, the present study highlights how a multiplicity of affective intensities are involved in maintaining a ‘provisional stability of action’ (Endrissat & Islam, 2022, p. 1029) in voluntary organising. In contrast to earlier studies, the findings show that in the public art project, voluntary organising is not propelled along a central axis of ‘affective dissonance’ (Marsh & Śliwa, 2022; Vachhani & Pullen, 2019, p. 29; Vidolov et al., 2023) but rather around the situational mobilisation of affects that prime different forms of support and compliance with artistic goals. Mobilising these different affective intensities allows voluntary organisations to coordinate heterogeneous individuals and groups who are not necessarily connected through a shared sphere of concern (Välikangas & Carlsen, 2020), and to navigate diverse interests and experiences in the organisational process. We can see in the findings how the four affective practices by coaxing bodies into affective encounters that elicit a sense of fascination, excitement, care or guilt forge sometimes fleeting sometimes more enduring ‘coalitional moments’ (Bruce & Homan, 2018, p. 226), in which collaborators’ roles, concerns and actions become aligned with the project’s agenda.

Enticing demonstrates how the crafting of encounters that are both pleasurable and bewildering invites participation by unsettling attachments to dominant norms and institutional ideals. The affective practice enriches recent work in organisation studies that discusses the power of affects such as bemusement (Vince, 2021) or fascination and awe in opening the self to ‘transubjective inspirations’ (Kenny & Fotaki, 2015, p. 189) that draw people towards new ways of relating. Envisioning underpins the affective organisational force of evocative imaginaries (Komporozos-Athanasiou & Fotaki, 2015) and elaborates on their capacity to light the ‘burning fire of desire’ (Hjorth, 2007, p. 720) that infects collaborators and spreads to new supporters. Resonant with a Butlerian sensibility for difference (Tyler, 2019), attending demonstrates how becoming responsive to others’ concerns and lifeworlds through recognition and compassionate validation of differences and interdependence facilitates convergence and commonality in voluntary organising, aiming for mutual feelings of care and respect rather than ‘harmony’ (van Marrewijk, Ybema, Smits, Clegg, & Pitsis, 2016, p. 1763). Asserting, meanwhile, underscores the argument of Sage et al. (2020, p. 353) that ‘inspiring sad passions’ by invoking concepts and ideas as worthy of protection is necessary for aligning and sustaining voluntary organising around new ideas and goals. This seems particularly relevant in contexts where collaborators are not likeminded, as is sometimes implicitly assumed (McCarthy & Glozer, 2022).

The findings challenge the idea of Munro and Thanem (2018) that voluntary organising can rely only on the engaging and coordinating effects of positive affect. Instead, the present study builds on and expands upon the observation of Vidolov et al. (2023, p. 20) that affective modes of organising rely on a ‘balanced dosing’ of positive and negative affect by showing how affectively engaging heterogeneous publics hinges on the situative evoking, compiling and balancing of different pleasurable and painful intensities that sustain a coalitional energy. In the organisational process, different affective practices are often paired to build and modulate intensities in interactions with stakeholders. Enticing and envisioning are combined in an attempt at moving fascinated publics towards more purposeful affective attachments. Attending is followed by enticing to open collaborators to a sense of curious wonder from a feeling of mutual respect, or asserting is combined with attending and envisioning to expand the restraining sense of guilt and frustration towards pleasurable feelings of being understood and excited so that participants do not become stuck in negative affects. Although this modulation of intensities strives towards cultivating and sustaining a ‘joyful’ (Munro & Thanem, 2018, p. 57) coalitional energy, it is not always successful. In the Bignik project, remnants of feelings of frustration and discomfort linger as intrusive affective traces in collaborative relations, prompting some people to distance themselves or take a break from the project as a means of protection. Thus, the affective coordination of voluntary organising through painful coalitional moments not only evokes dissonance as a productive force that moves and aligns people in participation (Vachhani, 2013; Vidolov et al., 2023) but also produces subtle forms of domination and infraction that cannot be fully erased by the seductive and exuberant intensities fostered around its aims. Future research should explore how affects such as awe or care can foster coalitional moments in other forms of voluntary organising, for instance, around sustainability initiatives, while considering more thoroughly how painful affects such as hate, fear or envy are elicited and fuel voluntary organising.

## Concluding Reflections

This paper contributes to theorising how voluntary organising can cultivate and sustain affective processes over time and across different settings by showing how affects are mobilised in patterns of activities that draw on materiality as an affect generator to project new organisational interests, promises and threats. Additionally, the study contributes to understanding how affective intensities coordinate and hold together voluntary organising by showing how the situative evoking, compiling and balancing of different positive and negative affects fosters a coalitional energy that continuously engages and aligns diverse individuals and groups with the agenda of the artistic project in the organisational process. Considering that we live in societies where values are increasingly organised through relationships, affective responses and concerns, affective practices might not only provide a conceptual tool to understand affective processes in voluntary organising, but also allow us to explore the mundane and subtle forms of affective labour that permeate other organisational domains, whether this is in innovation processes or in social, cultural and care work (Hardt & Negri, 2011). Given how prominently affects inform our experience of the present and our engagement in possible futures, it seems worthwhile to explore more broadly how diverse organisations practically enact, draw on and are grounded in the mobilisation of intensities such as hope, care, anxiety or frustration.

## Appendix

**Table 2.** Overview of the data structure of the four affective practices.

	Clustering and process	Ethnographic vignettes of affective practices	Interview and documentary traces of affective practices
Enticing	*affects*: intrigue/wonder/awe/fascination *activities*: staging surprising and inviting encounters/interactions around the art project *material driver*: sensuous and relational capacities of the fabrics (e.g. colour, texture, craft, playfulness) *intent*: pulling people out of their habituality into embracing and engaging with the art project	We are in a meeting with a tourism destination, discussing their involvement in the sewing process. Frank shows images of the sewing station and takes out a beautiful red module. Sarah marvels at it. He invites everyone to touch it. ‘It has a haptic quality. This one feels so silky; it’s probably a mixture of cotton and silk.’ Frank wants to set up a fabric collection station in their offices. Some seem a bit hesitant at first but then become charmed: ‘It is actually quite cool. Something different for a change’. (vignette 6) Fascinated, I watch how quickly the squares and streets are colouring in and coming alive around me as I help assemble the blanket. The farmer drives around boxes of modules on his small tractor and calls out if anyone needs more fabric. The artists put up an improvised sign indicating the ‘slipper station’. Pedestrians watch curiously and residents stare entranced out of their windows. People stop and join the process; some groups already sit down to have a picnic. (vignette 12)	‘I’m usually not the art type and alternative stuff like that is not truly my thing. However, for once, I like it . . . It looks very special from above because you just see this enormous blanket around a town or on a field . . . and that they keep going. That amazes me.’ (volunteer, field interview) ‘It was interesting when those who were also a bit resistant, if they stood on the Bignik because it was somewhere because they could do something, or sewed this part, then suddenly, they were all in.’ (partner, group interview) ‘I walked towards the train station and I see all these textiles. And voilà. I want to participate . . . I travel a lot, and abroad you always see that the tailoring and sewing takes place outside. In Switzerland, I have never seen that, and I found that tempting, that it takes place in the street, in the middle of everyday life.’ (participant, field interview)
Envisioning	*affects*: enthusiasm/excitement/anticipation/eagerness *activities*: evoking imaginaries that give meaning and significance to the artistic vision *material driver*: imaginative language (e.g. growing artwork, spilling/flooding streets, unorthodox solutions) *intent*: infecting people with a purposeful desire to materialise and be part of the art project	A woman approaches us at the sewing station: ‘What are you doing here?’ Frank explains that the project is running until 2043: ‘We are still at the beginning’ and gives her a flyer with the image of the blanket: ‘It is a growing artwork for the entire region.’ Her face lights up: ‘Oh this is great! Are you here tomorrow as well?’ The volunteer next to me and I are both excited by her eagerness: ‘It gives a special energy to be part of this big art project that takes place over so many years.’ (vignette 10) Showing images of an assembly in a town, the artists sketch the idea for a plan B if the fields are too wet: ‘We do an “asphalting” version, where we spill the blanket into the streets and alleys.’ Patrik draws a picture of a neighbour who wants to ‘flood his garden’. Members of the CDA nod animatedly: ‘It is great to be closer to the people. We need to be more unorthodox.’ A sense of anticipation inhabits the room as CDA and the artists come up with different ideas about how they could support the street assembly. (vignette 5)	‘You can do [the Bignik] only if people feel . . . a certain desire to work and sew on this vision and start to picture what it actually means if more and more modules are put together.’ (artist, documentation) ‘The idea of knotting fabric together, that is one thing. However, what it can trigger – the symbol – that is truly interesting.’ (partner, group interview) ‘Yes, it’s like . . . you just have to go sew, go lay out, if you can . . . It is like a virus, you almost can’t help it.’ (volunteer, group interview) ‘The artists have their own language and I catch myself adopting it into my vocabulary, and then I have to chuckle. At first, my reaction is often, “Well this is kind of semifunny” and afterwards I realise, it is actually spot on. When I read this “painting” of the town on Facebook for the first time, I thought, exactly that is what it is.’ (partner, group interview)
Attending	*affects*: care/respect/consideration/appreciation *activities*: exploring and accommodating concerns/perspectives of affected partners and stakeholders *material driver*: sharing practices that inhabit and give value to others’ lifeworlds (e.g. walking through the fields, doing a structured debriefing) *intent*: creating a mutual attunement that allows people to tolerate and accept aspects of the art project that are unsettling or inconvenient	The farmer arrives in boots and on a large machine, greeting the artists in a reserved manner. As we walk over to his field, Patrik inquires: ‘Over here you had a landslide, right? And you have three cattle. Where would you put them?’ The farmer points to an area. Patrik frowns: ‘Is that enough space?’ Frank comes with a map. The farmer follows closely as the artists discuss their ideas: ‘Would it be ok for you to mow this part?’ A smile flits across his face when they explain the shape of the blanket as an amorphous quadrat. ‘Ok, let’s have a look at what you need.’ As he walks briskly, the artists and I pick up his pace. (vignette 2) We meet the CDA at their new office for a debriefing. They look a bit weary. The artists compliment the space: ‘This calls for a celebration!’ Frank wants to know how the atmosphere is in the steering committee, with whom they have a tense relationship, and how CDA’s other major project is going. As the meeting starts, Frank pulls a series of documents out of his briefcase: ‘Let’s have a look at the agenda’. They discuss CDA’s concern about mobilising volunteers for the assembly, which they experience as a ‘burden’. Frank suggests working with fewer potential dates and organising a community meeting in advance. Lea appreciates the idea and seems relieved: ‘Who would we invite?’ (vignette 1)	‘When you have meetings with the artists, they are extremely present. One of the things that we would all like to be more. Also physically, when they enter a room, they are one of the few men who will hug other men . . . It’s great to work with them, they’re flexible . . . I didn’t believe that this fabric collection would work, but then, they just went exploring and talking to people time and again, visited them by train, by bus, on foot.’ (collaborator, group interview) ‘And they say “there are no problems, there are only solutions”. . . The way the artists dealt with such things, I liked that . . . because we always have differences and if you accept someone’s opinion, then they can suddenly also accept yours.’ (partner, interview) ‘I have always said that if you understand them, and if you understand their mechanics, if you understand how they work, then you can also influence them a little bit and everything will be ok. You have to be ready for that.’ (partner, group interview)
Asserting	*affects*: discomfort/frustration/guilt/disappointment *activities*: limiting ideas and maintaining boundaries around how the artwork is enacted *material driver*: affective power of core notions and experiences of the artistic idea (e.g. anticonsumerist space, value of collective contribution) *intent*: inhibiting people from pushing interests/ideas that undermine the artistic idea	At an impromptu meeting at the public sewing station, the artists discuss with the mayor the possibility of a tenth anniversary edition in the city, combined with sewing sessions and an exhibition at the textile museum. Sarah from the tourism destination suggests that people could eat in the museum. Frank isn’t thrilled. He gestures at the sewing station: ‘It is not the culinary that is the focus. It’s about connecting different people and the participative process. We had to teach that to CDA as well.’ She looks disappointed but quickly switches to empathising with the artist. ‘Of course’. (vignette 11) Frank is on the phone with a sewing machine manufacturer who feels that the partnership hasn’t been very fruitful and wants to rethink the collaboration. The partner asks whether they could buy a couple of modules for their own action. Frank negates firmly: ‘That is not possible. You couldn’t even pay for the value that these modules have. They are very precious.’ He suggests brainstorming ideas together in autumn. The partner is subdued and hesitates but agrees to discuss it internally, adding: ‘Maybe a private partnership is not the right instrument for the project.’ (vignette 3)	‘We have a responsibility vis-a-vis those fabrics. They are not ours; they belong to the community. The question of the ownership of this work will surface someday. [. . .] We cannot just rent it out like, “here you go, you can represent your merger [or business] with this blanket”. You can only become a part of the concept by lending a hand yourself.’ (artists, interview) ‘And I think the Riklins are like the guardians of the idea there and the idea is incredibly simple. However, you truly have to execute it to the end because, if you start compromising, then it falls apart at some point.’ (collaborator, group interview) ‘[With the artists] some things are all or nothing. There isn’t a little bit of Bignik; it is always the full charge and uncompromising . . . [t]hat probably also brings the success, I think.’ (partner, group interview) ‘And what I find exhausting, I sometimes notice it physically, when you want to question something, then you have a difficult stand . . . In this moment, you’re only met with resistance . . . It takes physical energy for me to face that.’ (collaborator, interview)
